# Health effects of the time-restricted eating in adults with obesity: A systematic review and meta-analysis

**DOI:** 10.3389/fnut.2023.1079250

**Published:** 2023-02-16

**Authors:** Weiyi Chen, Xiaoli Liu, Lei Bao, Ping Yang, Huihui Zhou

**Affiliations:** ^1^Department of Pathology, The Affiliated Yantai Yuhuangding Hospital of Qingdao University, Yantai, Shandong, China; ^2^Department of General Surgery, People’s Hospital of Rizhao, Rizhao, Shandong, China

**Keywords:** time-restricted eating, obesity, overweight, dietary interventions, meta-analysis

## Abstract

**Background:**

The number of people suffering from overweight or obesity has been steadily increasing in recent years. As a new form of diet, the efficacy of time-restricted eating (TRE) remains debatable.

**Objective:**

This meta-analysis quantified the effect of TRE on weight change and other physical parameters in obese and overweight adults.

**Methods:**

We did a systematic review and meta-analysis of randomized controlled trials (RCTs) comparing the TRE interventions on weight loss and other metabolic parameters by searching PubMed, Embase, and Cochrane Central Register of Controlled Trials to identify eligible trials published from database inception up until 23 August 2022. The risk of bias was assessed using the Revised Cochrane risk-of-bias tool (ROB-2.0). Meta-analysis was performed using Review Manager 5.4.1 software.

**Results:**

Nine RCTs with 665 individuals (345 in the TRE group while 320 in the control group) were included. Results indicated that TRE had a greater decrease in body weight (−1.28 kg; 95% CI [−2.05, −0.52], *p* = 0.001), fat mass (−0.72 kg; 95% CI [−1.40, −0.03], *p* = 0.04), body mass index (−0.34 kg/m^2^; 95% CI [−0.64, −0.04], *p* = 0.03) and diastolic blood pressure (−2.26 mmHg 95% CI [−4.02, −0.50], *p* = 0.01). However, the meta-analysis demonstrated that there was no significant difference between TRE and the control group in lean mass, systolic blood pressure, waist circumference, fasting glucose, fasting insulin, homeostasis model assessment-insulin resistance (HOMA-IR), total cholesterol, high-density lipoprotein, low-density lipoprotein, and triglycerides. Besides, the duration of the study and daily eating window also had an impact on weight change.

**Conclusion:**

TRE was associated with reductions in weight and fat mass and can be a dietary intervention option for adults with obesity. But high-quality trials and longer follow-ups are needed to draw definitive conclusions.

## 1. Introduction

Obesity has reached epidemic proportions around the world, with approximately 39% of adults classified as overweight and more than 600 million classified as clinically obese by 2020 (1). Considered an epidemic and, consequently, a public health problem, it is not only directly associated with non-communicable diseases and chronic diseases, such as diabetes mellitus, cardiovascular diseases, brain stroke, certain cancers, obstructive sleep apnea, and osteoarthritis, but also has important consequences for disability, emotional wellbeing, and quality of life (2, 3).

Some studies have observed an association between weight loss and improvement in some cardiometabolic markers such as serum triglycerides and cholesterol, free fatty acids, leptinemia, glucose, insulinemia, and blood pressure (4–8). Body weight and fat mass are regulated by many physiological mechanisms, energy imbalance due to increased caloric intake and reduced physical activity is one of the major causes of obesity in adults (9). Lifestyle interventions, including qualitative and quantitative nutritional changes, as well as increased exercise, have been the first line of treatment for obesity and metabolic diseases. However, body weight is regulated by numerous physiological mechanisms, far beyond voluntary food intake, and physical exercise. When a person loses weight the body fights back, with physiological adaptations on both sides of the energy balance equation that try to bring body weight back to its original state (10, 11).

Surrounded by highly palatable and energy-dense processed foods, many people tend to consume more energy than they burn, making it difficult to achieve sustained clinically significant weight loss by long-term calorie restriction (12). Treatment of obesity is multidisciplinary, with lifestyle changes being the first option, including changes in food choices and increased levels of physical activity (13). The investigation of dietary approaches that may promote patient adherence to treatment is a fruitful area of research (14).

As reported in the review of the literature in 2020, intermittent fasting which is a dietary pattern based upon timed periods of fasting, is beneficial in preclinical and clinical studies in a variety of conditions like obesity, diabetes, heart disease, cancer, as well as neurological disorders (15). Given the various options for intermittent fasting, time-restricted eating (TRE) has gained scientific attention in recent years. This approach proposes a fasting period of 8–12 h/day, followed by a period of free eating or eating associated with energy restriction (16). The method has gained popularity because it is a simple and easy weight loss strategy, which may improve adherence rates (17). TRE prevents weight gain in mice with a high-fat, isocaloric diet (18), and reduces body weight and metabolic results in mice that are already obese (19). In humans, there is a growing number of studies in different fields involving TRE recently.

Since high BMI is a high-risk factor for multiple health problems, the purpose of this article is to assess the effect of TRE studies on changes in body weight and fat mass (primary outcome) and changes in other anthropometric and metabolic variables (secondary outcomes) in adults with overweight or obesity. We used standard cutoff points of BMI to define overweight (BMI, 25–29) and obesity (BMI, ≥30).

## 2. Materials and methods

### 2.1. Protocol and registration

**FIGURE 1:**
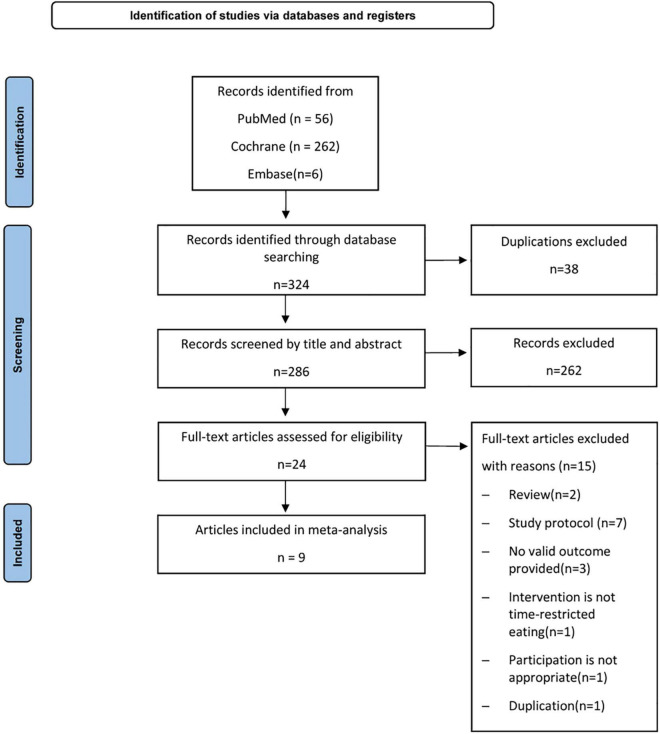
PRISMA (Preferred Reporting Items for Systematic Reviews and Meta-Analyses) flow diagram.

The systematic review was conducted as per PRISMA (Preferred Reporting Items for Systematic Reviews and Meta-Analyses) guidelines (20). Figure 1 shows the pattern of study selection.

The protocol has been registered at Prospero: CRD42022361240.

### 2.2. Inclusion and exclusion criteria

Only randomized controlled trials (RCTs) that met the following criteria were included: the study participants were, 18 years old and above with a body mass index (BMI) greater than 25 kg/m^2^. The intervention of the experimental group was TRE in which all participants were restricted to eating within an eating window from 8 to 12 h while the control group was not restricted by diet time. The participants were allowed to eat *ad libitum* or follow a hypocaloric diet as long as they followed the same diet in the same trial. There were no restrictions based on sex, race, or country.

The exclusion criteria involved studies (a) not RCT, (b) combined with other interventions, (c) participants with diseases impacting on outcomes, (d) without quantitative outcomes, and (e) duplicate publications.

### 2.3. Search strategy

We searched PubMed (National Library of Medicine), Embase, and Cochrane Central Register of Controlled Clinical Trials for studies published from inception to 23 August 2022, following the PICO (participants, interventions, comparisons, outcomes) principles. We also searched the gray literature on ClinicalTrials.gov, OpenGrey.eu, and Greylit to reduce publication bias. There was no language restriction.

The Medical Subject Headings (MeSH) along with keyword terms utilized were “fasting” or “Intermittent Fasting” or “time-restricted feeding” or “time-restricted eating” and “overweight” or “obesity” or “obese” and “adult” and “random” or “trial.”

### 2.4. Study selection and data extraction

Two authors (WC and LB) independently screened the titles and abstracts of the publications identified in the search and relevant articles were retrieved as full texts. If there were different opinions on the inclusion or exclusion of studies, a third author (HZ) would contribute to the discussion and arrive at a consensus result. Where there was missing data, we contacted the authors for additional information. If data is not shown in the text but is available in the Supplementary material, it will be extracted in the Supplementary material but we give priority to the content of the text.

Where multiple analyses (intention to treat or per-protocol) were reported by the authors, more conservative analyses of intention to treat were extracted, but where the abandonment rate exceeded 45%, protocol analyses were used (21). Besides, when different analysis methods were used for the text and the Supplementary material, we gave preference to the analysis method of the text (22).

Two authors (WC and XL) extracted data independently *via* Microsoft Excel 2021. One author (HZ) supervised the selection along with the data abstraction process. The following information was collected from each included study (1) first author name and year of publication; (2) age; (3) baseline BMI; (4) the number of individuals enrolled in each group; (5) duration of eating window; (6) study duration; (7) diet restriction; (8) outcome measurement; (9) study attrition; and (10) the following human variation parameters: weight, BMI, fat mass, lean mass, systolic blood pressure (SBP), diastolic blood pressure (DBP), total cholesterol, triglycerides, high-density lipoprotein (HDL), low-density lipoprotein (LDL), fasting glucose, fasting insulin, homeostasis model assessment-insulin resistance (HOMA-IR), and waist circumference (WC).

### 2.5. Study risk of bias assessment

We used the “Revised Cochrane risk-of-bias tool for randomized trials (ROB-2.0)” to assess the quality of RCTs (23). Bias was assessed as a judgment (high, low, or unclear) for elements from five domains: (1) randomization process; (2) deviations from intended interventions; (3) missing outcome data; (4) measurement of the outcome; and (5) selection of the reported result. All the authors independently participated in the quality assessment and agreed with the results.

### 2.6. Statistical analysis

Both quantitative synthesis and subgroup analysis were performed with Review Manager version 5.4.1 (24). The pooled effect sizes were expressed as mean difference (MD) with a 95% confidence interval (95% CI). *p* < 0.05 indicates a statistical significance. If the heterogeneity was relatively low (*I*^2^ < 50%), we used a fixed effects model, otherwise a random effects model was applied. For the analysis of all parameters, we use the change between the end of intervention and baseline. If the standard deviation of the change from the baseline is not given in the original research, we assumed an intraparticipant correlation of 0.5 from baseline to follow-up measurements to calculate missing values according to the recommendations in the Cochrane Handbook.

**FIGURE 2:**
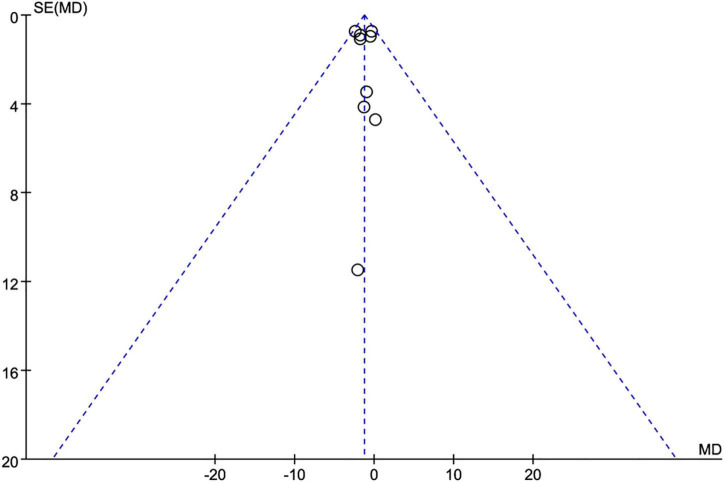
Funnel plot for publication bias detection on weight loss changes. MD, mean difference; SE, standard error.

Meanwhile, the subgroup analysis was conducted according to the duration of the eating window (8 vs. >8 h), study duration (<12 vs. ≥12 weeks), and energy intake (with restriction or eat *ad libitum*) in participants. Publication bias was examined with a funnel plot asymmetry (Figure 2) and Egger’s test. The results of at least four studies were analyzed for data synthesis.

## 3. Results

### 3.1. Study selection

The literature search yielded 324 articles and excluded 38 duplicate studies from the beginning until 23 August 2022. A total of 286 studies were available for a review of titles and abstracts, of which 24 were fully reviewed. A total of 15 studies were excluded 2 reviews, 7 protocols, 3 studies without valid outcomes, 2 studies with inappropriate intervention or participants, and 1 duplication as shown in Figure 1. Nine studies (21, 22, 25–31) (665 participants) met the inclusion criteria and were identified and underwent systematic review and data synthesis.

### 3.2.Study characteristics

All included studies were conducted on adults aged 18 years and above whose BMI was more than 25 kg/m^2^. The largest study enrolled 139 participants (27) whereas the smallest 20 participants (31). Only the duration of the eating window differed between the TRE and control groups in the same study, the rest of the interventions (e.g., exercise and calorie restriction) were the same. Participants in three studies (26, 29, 30) were allowed to eat *ad libitum*. Participants in six studies (21, 22, 25, 27, 28, 31) were advised to follow a calorie-restricted diet, one of which was to be combined with exercise (28). All the studies included an intervention group with a TRE duration of 8 h (22, 26–30), 10 h (25, 31), and 12 h (21). For the control groups, the eating window was ≤12 h (22, 28, 31) or with no restriction (21, 25–27, 29, 30). The duration of the intervention was 8 weeks (22, 29, 31), 12 weeks (26, 30), 14 weeks (28), 39 weeks (25), and 12 months (21, 27). The results of one study (31) were measured by the participants themselves at home, and the rest were objectively measured.

The characteristics of the literature chosen for quantitative synthesis are listed in Table 1.

**TABLE 1:** General features of the nine included articles.

References	Country	Age	BMI	Sample size		Duration of eating windows		Study duration	Diet restriction
				TRE group	Control group	TRE group	Control group		
Isenmann et al. (29)	Germany	20–40	25–33	18 (10F)	17 (11F)	8 h	No restriction	8 weeks	The participants were allowed to eat *ad libitum*
Chow et al. (30)	USA	45.5 ± 12.1	≥25	11 (9F)	9 (8F)	8 h	No restriction	12 weeks	The participants were allowed to eat *ad libitum*
Jamshed et al. (28)	USA	43 ± 11	39.6 ± 6.7	45 (35F)	45 (37F)	8 h	≥12 h	14 weeks	The participants were counseled to follow a hypocaloric diet (500 kcal/day below their resting energy expenditure) and exercise 75–150 min/week
Liu et al. (27)	China	31.9 ± 9.0	28–45	69 (33F)	70 (35F)	8 h	No restriction	12 months	All the participants were instructed to follow a calorie-restricted diet that consisted of 1,500–1,800 kcal/day for men and 1,200–1,500 kcal/day for women
Thomas et al. (25)	USA	38.0 ± 7.8	34.1 ± 5.7	41 (34F)	40 (35F)	10 h	No restriction	39 weeks	Caloric restriction for both groups
de Oliveira Maranhão Pureza et al. (21)	Brazil	19–44	33.3 ± 4.1	31F	27F	12 h	No restriction	12 months	All the participants were instructed to follow a hypo-energetic diet
Lowe et al. (26)	USA	46.5 ± 10.5	32.7 ± 4.2	59 (24F)	57 (22F)	8 h	No restriction	12 weeks	The participants were allowed to eat *ad libitum*
Peeke et al. (31)	USA	18–65	≥30	39	39	10 h	12 h	8 weeks	Both groups were reduced in energy relative to expenditure for baseline body weight (approximately 500–1,000 kcal/day deficit)
Queiroz et al. (22)	Brazil	30 ± 6	30.5 ± 2.7	32	16	8 h	12 h	8 weeks	Participants were prescribed a diet plan to promote weight loss, but no food was provided. Energy intake was calculated based on each individual resting metabolic rate multiplied by the physical activity levels of 1.4, −25% of the daily energy requirements
References		Outcome measurement						Study attrition	
Isenmann et al. (29)		Weight and anthropometric parameters were measured objectively four times (at the beginning and end of the familiarization phase; the end of the intervention; 6 weeks after the intervention).						42 participants 7 dropped out at the familiarization phase, 35 completed the study	
Chow et al. (30)		Body weight, composition, and metabolic outcomes were measured pre and end-intervention objectively.						22 participants, 20 completed	
Jamshed et al. (28)		Bodyweight was measured in the non-fasting state in the clinic every 2 weeks throughout the trial. Additional outcomes were measured at week 0 and week 14 objectively.						90 participants, 59 completed	
Liu et al. (27)		The outcomes were quantified objectively at baseline and 12 months.						139 participants, 118 completed	
Thomas et al. (25)		Objective clinic weights and body composition measurements were obtained at baseline and 39 weeks.^a^						81 participants, 63 completed	
de Oliveira Maranhão Pureza et al. (21)		The results were measured before and after, 4, 6, and, 12 months of intervention objectively.						58 participants, 27 completed	
Lowe et al. (26)		All participants had their weight measured at home using a Bluetooth scale, which was linked to the research platform. 46 participants completed extensive in-person metabolic testing in Clinical Research Center.						116 participants, 105 completed	
Peeke et al. (31)		Study supplies (scale, glucometer, lancets, and glucose strips) were shipped to the participant’s homes, and study procedures and assessments were conducted by participants at home.						78 participants, 60 completed	
Queiroz et al. (22)		Body weight and anthropometric outcomes were measured in the laboratory by the same experienced researchers.						48 participants, 37 completed	

Mean ± SD, range; F, female. ^a^Home scale weights were obtained in one cohort (*n* = 26) from week 6 to week 12.

### 3.3. Risk of bias assessment

**FIGURE 3:**
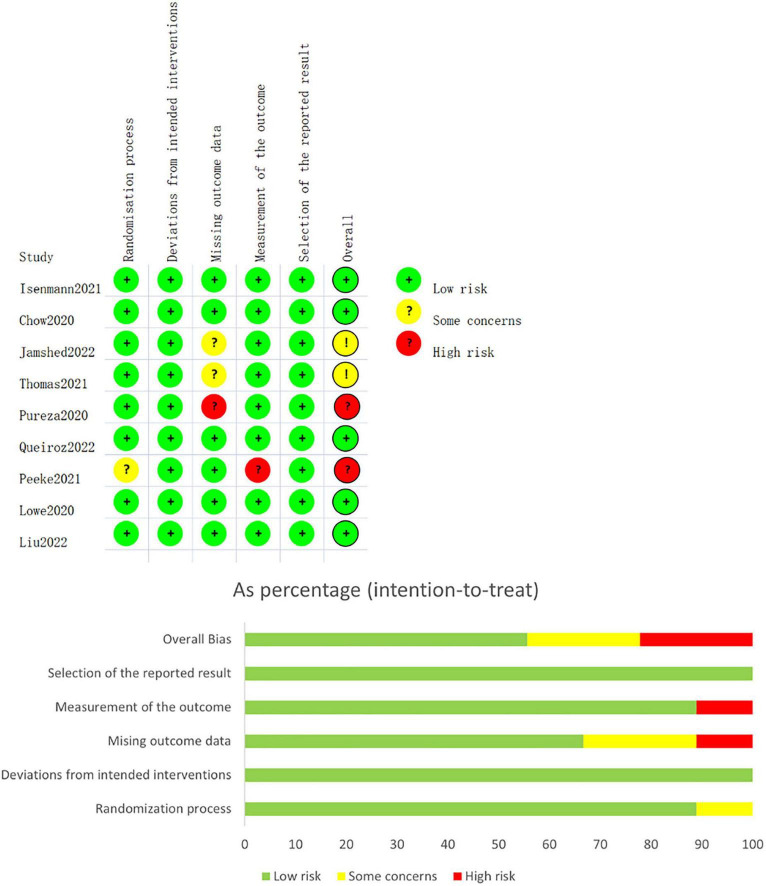
Risk of bias assessment.

A graphic summarizing the risk of bias was produced from discussions among the authors, as shown in Figure 3. Five studies (22, 26, 27, 29, 30) had low risk of bias. Two studies (25, 28) had some concerns of bias risk due to missing data from moderate dropout rates. One study (21) had high risk of bias due to high dropout rate (53.4%) from long intervention period. One study (31) was categorized as having a high risk of measurement of the outcome because the study procedures and assessments were conducted by participants at home. The study also had some concerns about the risk of bias in random processes, because an equivalent number of men were assigned to each group.

### 3.4. Efficacy

#### 3.4.1. Weight and BMI

**FIGURE 4:**
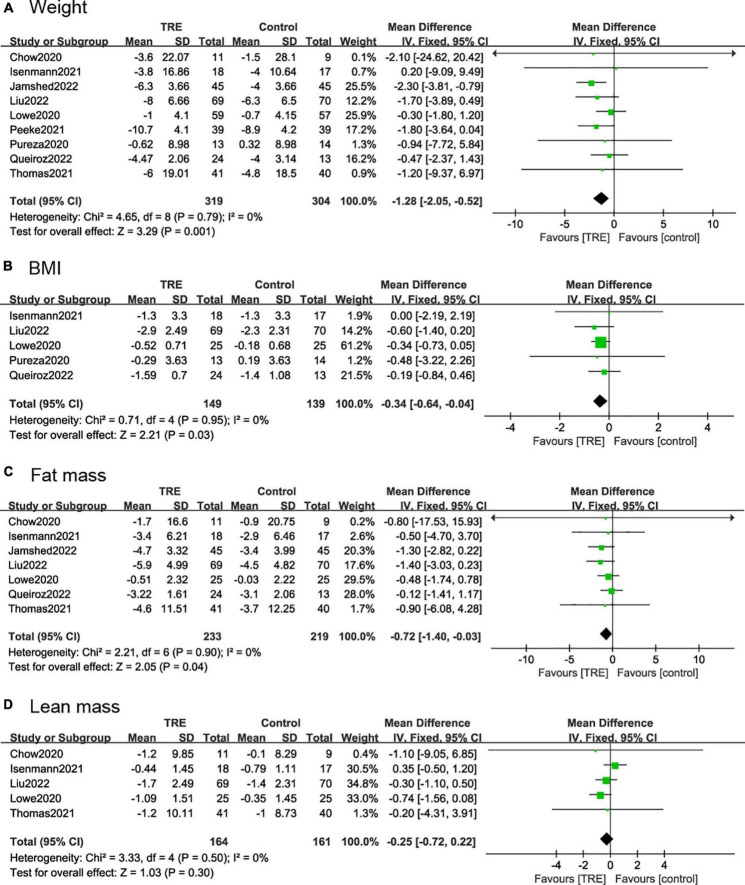
Forest plots of comparisons between TRE and the control groups in **(A)** weight; **(B)** body mass index (BMI); **(C)** fat mass; and **(D)** lean mass.

Nine studies (21, 22, 25–31) (623 individuals, 319 in the TRE group, 304 in the control group) analyzed weight as an outcome. Individuals assigned to the TRE intervention group showed a significant weight reduction compared to the control group (−1.28 kg; 95% CI [−2.05, −0.52]; *p* = 0.001; *I*^2^ = 0%) (Figure 4A). No significant publication bias was detected by Egger’s test (*p* = 0.543).

Five studies (21, 22, 26, 27, 29) (288 individuals, 149 in the TRE group, 139 in the control group) analyzed BMI as an outcome. Participants allocated to the TRE group showed a significant reduction in BMI compared to the control group (−0.34 kg/m^2^; 95% CI [−0.64, 0.04]; *p* = 0.03; *I*^2^ = 0%) (Figure 4B).

#### 3.4.2. Fat mass and lean mass

Seven studies (22, 25–30) included fat mass as an outcome, with 452 individuals (233 in the TRE group, 219 in the control group) evaluated. It demonstrated that the TRE group showed a slight difference in fat mass compared to the control group (−0.72 kg; 95% CI [1.40, −0.03], *p* = 0.04, *I*^2^ = 0%) (Figure 4C).

Five studies (25–27, 29, 30) included lean mass as an outcome with 325 individuals (164 in the TRE group, 161 in the control group) evaluated. It demonstrated that there was no difference in lean mass between groups (−0.25 kg; 95% CI [−0.72, 0.22], *p* = 0.30; *I*^2^ = 0%) (Figure 4D).

#### 3.4.3. Blood pressure

**FIGURE 5:**
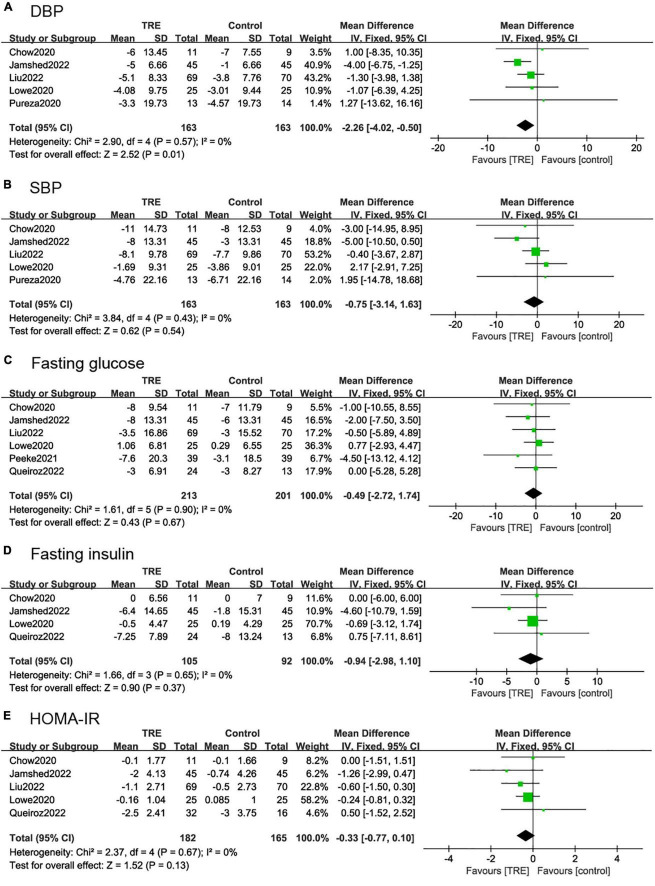
Forest plots of comparisons between TRE and the control groups in **(A)** diastolic blood pressure (DBP), **(B)** systolic blood pressure (SBP), **(C)** fasting glucose, **(D)** fasting insulin, and **(E)** homeostasis model assessment-insulin resistance (HOMA-IR).

Five studies (21, 26–28, 30) (326 individuals, 163 in the TRE group, and 163 in the control group) analyzed SBP and DBP as the outcome. The TRE group showed a statistically significant reduction in DBP (−2.26 mmHg; 95% CI [−4.02, −0.50], *p* = 0.01, *I*^2^ = 0%) compared to the control group, however, there was no difference in SBP (−0.75 mmHg; 95% CI [−3.14, 1.63], *p* = 0.54, *I*^2^ = 0%) (Figures 5A, B).

#### 3.4.4. Fasting glucose, insulin, and HOMA-IR

Six studies (22, 26–28, 30, 31) (364 individuals, 213 in the TRE group, 201 in the control group) tested fasting glucose for results. It demonstrated that there was no difference in fasting glucose levels between groups (−0.49 mg/dl; 95% CI [−2.72, 1.74], *p* = 0.67, *I*^2^ = 0%) (Figure 5C).

Five studies (22, 26–28, 30) (197 individuals, 105 in the TRE group, 92 in the control group) analyzed fasting insulin as an outcome. Individuals in the TRE group showed no differences in fasting insulin levels compared to the control group (−0.94 mU/L; 95% CI [−2.98, 1.10], *p* = 0.37 *I*^2^ = 0%) (Figure 5D).

Five studies (22, 26–28, 30) (347 individuals, 182 in the TRE group, 165 in the control group) analyzed HOMA-IR as an outcome. It demonstrated that there was no difference in HOMA-IR between groups (−0.33; 95% CI [−0.77, 0.10], *p* = 0.13, *I*^2^ = 0%) (Figure 5E).

#### 3.4.5. Total cholesterol and triglycerides

**FIGURE 6:**
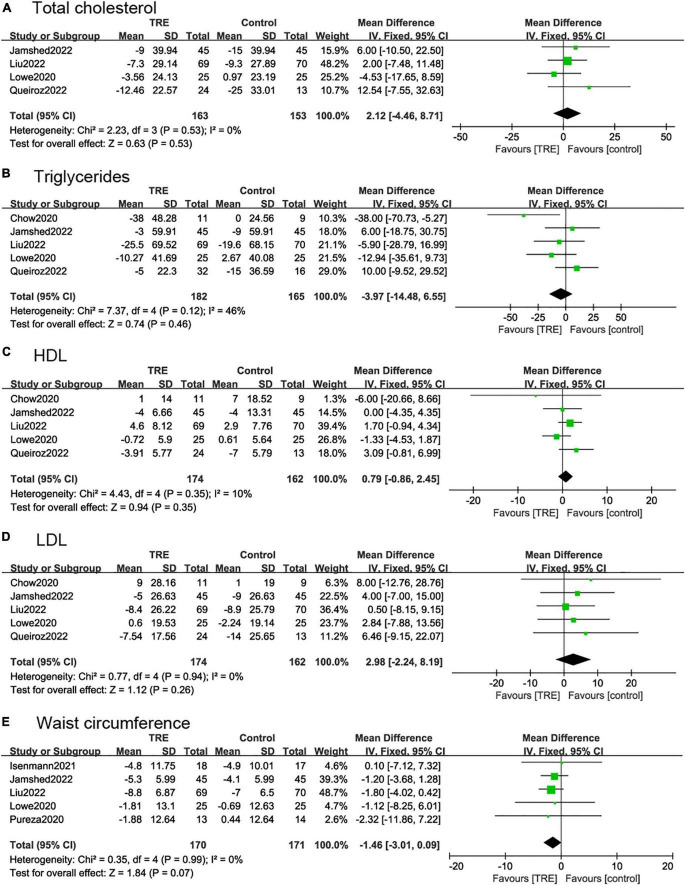
Forest plots of comparisons between TRE and the control groups in **(A)** total cholesterol; **(B)** triglycerides; **(C)** high-density lipoprotein (HDL); **(D)** low-density lipoprotein (LDL); and **(E)** waist circumference.

Four studies (22, 26–28) (316 individuals, 163 in the TRE group, 153 in the control group) analyzed total cholesterol as an outcome. Individuals in the TRE group did not show differences in total cholesterol compared to the control group (2.12 mg/dl; 95% CI [−4.46, 8.71], *p* = 0.53, *I*^2^ = 0%) (Figure 6A).

Five studies (22, 26–28, 30) (347 individuals, 182 in the TRE group, 165 in the control group) analyzed triglycerides as an outcome. Individuals in the TRE group showed no differences in triglyceride levels compared to the control group (−3.97 mg/dl; 95% CI [−14.48, 6.55] *p* = 0.46, *I*^2^ = 46%). In sensitivity analyses, when removing the study by Chow et al. (30), the heterogeneity decreased to *I*^2^ = 0%, however, the results were still not statistically significant (−0.05 mg/dl; 95% CI [−11.06, 11.05] *p* = 0.99 *I*^2^ = 0%) (Figure 6B).

#### 3.4.6. HDL and LDL

Five studies (22, 26–28, 30) reported HDL and LDL, with 336 individuals (174 in the TRE group, 162 in the control group) evaluated. The results demonstrated that there was no statistical difference between TRE and control groups in terms of HDL (0.79 mg/dl; 95% CI [−0.86, 2.45], *p* = 0.35, *I*^2^ = 0%) and LDL (2.98 mg/dl; 95% CI [−2.24, 8.19], *p* = 0.26, *I*^2^ = 0%) (Figures 6C, D).

#### 3.4.7. Waist circumference

Five studies (21, 26–29) (341 individuals, 170 in the TRE group, 171 in the control group) analyzed WC as an outcome. These demonstrated that TRE had a small effect on WC but with no statistical difference compared with control groups (−1.46 cm; 95% CI [−3.01, 0.09], *p* = 0.07, *I*^2^ = 0%) (Figure 6E).

### 3.5. Subgroup analysis

#### 3.5.1. Eating window

**FIGURE 7:**
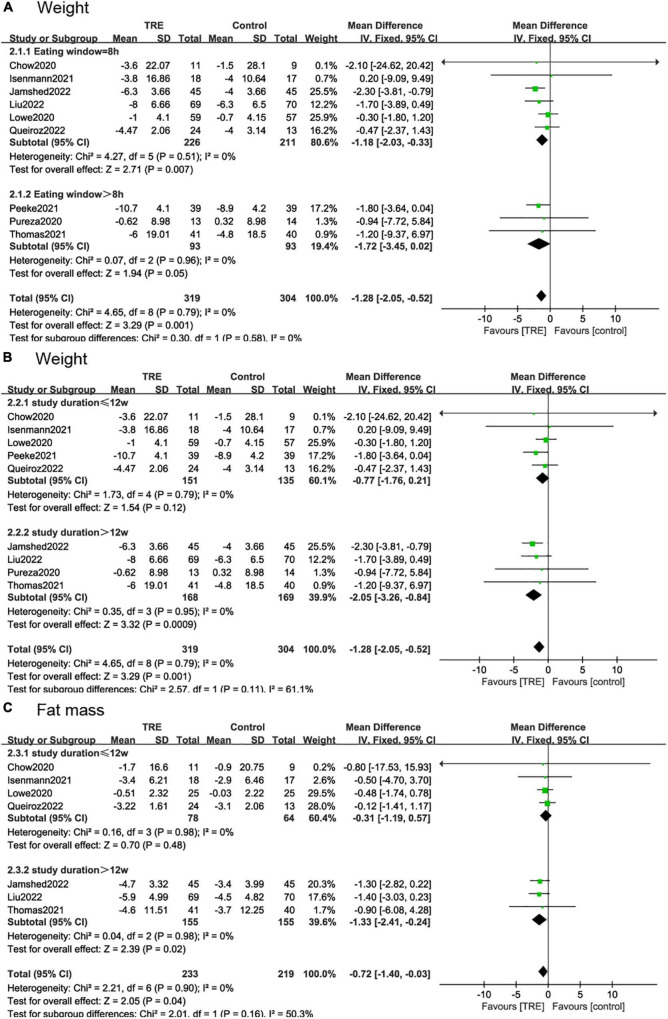
Forest plot of **(A)** weight change under the eating window in subgroups of 8 h (2.1.1) and over-8 h (2.1.2); **(B)** weight change under the study durations in subgroups of ≤12 weeks (2.2.1) and >12 weeks (2.2.2); and **(C)** fat mass change under the study durations in subgroups of ≤12 weeks (2.3.1) and >12 weeks (2.3.2).

According to the duration of the eating window, we divided the studies into two subgroups, 8 h (22, 26–30) and over 8 h (21, 25, 31). The 8-h eating window showed a significant weight reduction (−1.18 kg; 95% CI [−2.03, −0.33], *p* = 0.007, *I*^2^ = 0%) compared to the control group while over 8-h eating window showed no statistical difference (−1.72 kg; 95% CI [−3.45, 0.02], *p* = 0.05, *I*^2^ = 0%) (Figure 7A).

#### 3.5.2. Study duration

In the subgroup analysis for the study duration, we divided the studies into two subgroups. In studies ≤12 weeks (22, 26, 29–31), weight change (−0.77 kg; 95% CI [−1.76, 0.21], *p* = 0.12, *I*^2^ = 0%) and fat mass reduction (−0.31 kg, 95% CI [−1.19, 0.57], *p* = 0.48, *I*^2^ = 0%) showed no difference in the TRE group compared to the control group. However, in studies >12 weeks (21, 25, 27, 28), these two indicators showed a statistically significant difference, with weight (−2.05 kg; 95% CI [−3.26, −0.84], *p* = 0.0009, *I*^2^ = 0%) and fat mass (−1.33 kg; 95% CI [−2.41, −0.24], *p* = 0.02, *I*^2^ = 0%) (Figures 7B, C).

#### 3.5.3. Energy restriction

**FIGURE 8:**
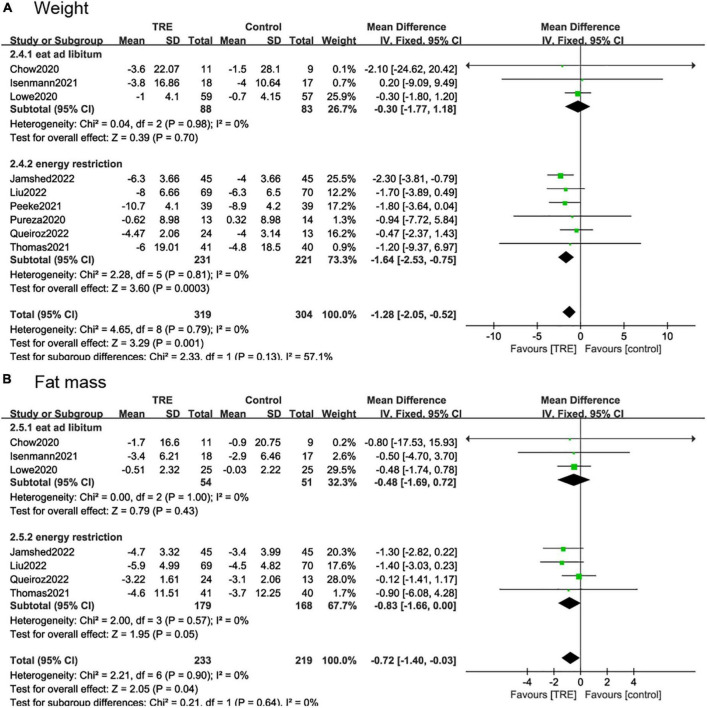
Forest plot of **(A)** weight change in subgroups of participants allowed to eat libitum (2.4.1) and participates with caloric restriction (2.4.2); and **(B)** fat mass change in subgroups of participants allowed to eat libitum (2.5.1) and participates with caloric restriction (2.5.2).

We divided the study into two subgroups based on whether there were energy restrictions on the participants. In studies where participants were allowed to eat libitum (26, 29, 30), and there was no difference in weight (−0.3 kg; 95% CI [−1.77, 1.18], *p* = 0.70, *I*^2^ = 0%) and fat mass (−0.48 kg; 95% CI [−1.69, 0.72], *p* = 0.43, *I*^2^ = 0%) between the TRE and the control group. In the studies with caloric restriction (21, 22, 25, 27, 28, 31), there was a significant weight reduction (−1.64 kg; 95% CI [−2.53, −0.75], *p* = 0.0003, *I*^2^ = 0%) in the TRE group compared to the control group. In terms of fat mass, the TRE group also showed a beneficial effect (−0.83 kg; 95% CI [−1.66, −0.00], *p* = 0.05, *I*^2^ = 0%) (Figures 8A, B).

### 3.6. Adverse events

One study (27) reported mild adverse events such as fatigue, dizziness, headache, decreased appetite, upper abdominal pain, dyspepsia, and constipation, but the occurrences were similar in TRE and control groups. Hunger and headaches were observed in the TRE group in one study in the first few weeks, but tended to disappear over time (22).

Time-restricted eating regimen was reported to affect daily physical activities and psychological conditions. In one study, a significant reduction in daily movement and step count were observed in the TRE group (26). But in another study, there were no between-group differences in self-reported physical activity and the TRE intervention was more effective at improving total mood disturbances (28).

## 4. Discussion

The present review and meta-analysis included nine studies, with 665 individuals in total. We found a significant decrease in body weight, fat mass, BMI, and DBP compared to the control group. However, there were no changes in lean mass, SBP, WC, insulin, glucose, HOMA-IR, and lipid profile (triacylglycerol, HDL, LDL, and total cholesterol). Although an article on the impact of TRE on people with obesity has been published before (32), several high-quality RCTs addressing the effects of TRE on obese populations emerged later (22, 25, 27, 28), and some are on the way, therefore, this study was necessary.

Through subgroup analysis, we found that the duration of the study had a significant effect on weight change as well as fat mass. In studies longer than 12 weeks, weight change (−2.05 kg; 95% CI [−3.26, −0.84], *p* = 0.0009, *I*^2^ = 0%) and fat mass reduction (−1.33 kg, 95% CI [−2.41, −0.24], *p* = 0.02, *I*^2^ = 0%) were more significant in the TRE group compared to the control group. And in studies less than 12 weeks, those two outcomes did show no difference in the TRE and control groups. However, in a previous meta-analysis (32), the results were that short-term TRE intervention was more effective, which is contrary to the findings of this study. This may be due to the emergence of more RCTs with more participants.

In a subgroup analysis on the duration of the eating window, we also found out that the duration of the eating window was essential to the effect of weight. The 8-h eating window was more effective in weight reduction (−1.18 kg; 95% CI, [−2.03, −0.33], *p* = 0.007, *I*^2^ = 0%) compared with the control group. The over-8 h eating window was also more TRE favorable, but it was not statistically different compared to the control group (−1.72 kg; 95% CI [−3.45, 0.02], *p* = 0.05, *I*^2^ = 0%). This indicates that it would be more useful if limit the length of eating time in the TRE method. Excessive daily eating time will reduce the effect of weight loss. However, compressing the diet to less than 8 h will make adherence more difficult and reduce compliance over the long-term, due to occasional social needs in the evening, which may prolong the eating time. Achieving a balance between improved adherence and good results is the key to following TRE.

In the subgroup analysis targeting energy intake, we found that the studies with caloric restriction (21, 22, 25, 27, 28, 31) had a significant benefit in both weight and fat loss in the TRE group compared to the control group. But in trials (26, 29, 30) where participants were allowed to eat *ad libitum*, there was no statistical difference in weight change. However, due to the low proportion of participants who eat *ad libitum* (26.6% on weight analysis and 23.2% on fat mass analysis), caution is needed when recommending TRE combined with energy restriction.

Time-restricted eating has been approved to be effective in preventing obesity and improving metabolic outcomes in several animal models of obesity. Mice under TRE (food access restricted to 8–10 h) consume equivalent calories as those with *ad libitum* access yet were protected from excessive weight, hyperinsulinemia, inflammation, and hepatic steatosis and have improved motor coordination (18, 33). Rats under TRE also had lower weight gain and adiposity than those on the matched *ad libitum* diet (34). Remarkably, a study by Chaix et al. (35) showed that most of the benefits of TRE are sex-dependent, the TRE prevents weight gain only in male mice. For humans, it appears that men are more likely to lose weight than women in weight loss attempts (36). In the nine trials we included, there was a preponderance of female participants but no gender-specific analysis was given. Further studies are needed to determine the effects of TRE among men and women separately.

Some human observational studies have shown that more than 50% of adults eat for 15 h or more each day, and they often consume most of their calories later in the day (37, 38). A prospective longitudinal study of 420 overweight patients indicated that people who ate late for lunch lost significantly more weight than those who ate early, pointing to the importance of the time of day when food is consumed (39). Based on these considerations, TRE, a relatively new approach to weight management that has emerged and been the focus of attention for both lay people and scientists in recent years, has proven to be a beneficial strategy for inducing weight loss because it can maintain a consistent daily cycle of eating and fasting that supports circadian rhythms (37). TRE did not need to deliberately instruct participants to limit their total energy intake because there was no intentional energy restriction during TRE, but only a change in the timing of eating, which greatly reduced the difficulty of adherence.

Time-restricted eating is a more reasonable and feasible approach than calorie restriction because there is no deliberate energy restriction, only a change in the timing of the diet. In our pool of studies, the adherence rate averages above 70%, but varies relatively widely, which is the main source of bias, from 46.5% (40) to 90.9% (30). The high dropout rate may be due to the long duration of the experiment and financial reasons (40). And the reason for high adherence is that only participants who demonstrated high adherence during the pre-intervention period were included (30).

We observed that weight loss was accompanied by a decrease in lean body mass in both the TRE and control groups. Loss of lean mass can lead to rapid weight regain as well as many health problems, including the impact on health, the ability to perform activities of daily living, and the potential impact on the emotional and psychological state (38, 41). Losing weight while maintaining lean body mass would be ideal. Exercise has been shown to help offset some of the changes in lean mass experienced with weight loss (42, 43). In addition, augmented protein intake, and dietary supplements such as chromium picolinate are proven to help preserve lean mass during weight loss (44).

Greater adherence to some proven healthy diets such as the Mediterranean diet has been associated with significant improvements in health status (45, 46). The RCTs we included are those with unlimited diets and calorie restrictions but there are currently no RCTs combined with specific healthy eating practices. If TRE is combined with a specific healthy diet, it will be interesting to see how it affects weight loss and reduces the risk of metabolic and cardiovascular disease.

In individuals without overweight or obesity, isoenergetic TRE was related to higher fasting glucose and a bigger impairment of glucose tolerance (47). In people with obesity (48) or metabolic syndrome (6), TRE with *ad libitum* intake did not change fasting glucose, fasting triglycerides, or HOMA-IR. In the healthy individuals, TRE did not show weight loss, but still showed beneficial effects like fat mass reduction (49) which indicates that TRE could be beneficial to the population with or without metabolic dysfunction.

More than half of the trials included in this systematic review were conducted in the US (25, 26, 28, 30, 31). These populations may have different dietary patterns compared with those from other countries, which may contribute to a potential risk of bias. Retaining participants for long-term lifestyle interventions can be difficult and bias is a concern when high dropout rates occur. In addition, all trials included did not analyze males and females separately, and we cannot know whether TRE differs between genders. We recommend more high-quality RCTs conducted in different countries as well as genders. Furthermore, only two studies (22, 27) reported mild adverse events. It is unclear whether adverse events occurred in other studies. We strongly suggest that future human studies take possible adverse events into account.

This meta-analysis included nine RCTs and concentrated on the efficacy of TRE in adults with obesity. In comparison with previous studies, we included some new RCTs and add some outcome indicators. Many limitations of this study should be recognized. Firstly, the number of RCTs is insufficient, the sample size is insufficient, and some studies have a high risk of bias. Secondly, the majority of participants were women, making the results difficult to generalize. Thirdly, most of the studies have a short duration, we cannot know long-term benefits and safety. Finally, the intention-to-treat analyses of RCTs may lead to relatively conservative results. There are still some unfinished experiments on ClinicalTrials.gov (50–52) and when these are completed and included in the meta-analysis, a more definitive conclusion will be drawn.

## 5. Conclusion

We concluded that the TRE regimen seems to have a beneficial effect on weight and fat mass reduction, and improves BMI and DBP, but no significant effects on other metabolic parameters were observed. Subgroup analysis showed that the eating window should not be too long, with better results below 8 h. In addition, TRE combined with calorie restriction may have a better effect, but caution is needed due to the insufficient sample. It is unclear if TRE has the same effect on males and females. We strongly recommend that future human studies take gender issues into account and analyze them separately. Further high-quality RCTs and longer follow-up studies are needed to make clearer conclusions.

## Author contributions

WC and LB: conceptualization and formal analysis. HZ: methodology, validation, supervision, and funding acquisition. XL: software and investigation. LB: resources. WC and XL: data curation and writing—original draft preparation. WC and PY: writing—review and editing. PY: visualization. WC: project administration. All authors had read and agreed to the published version of the manuscript.
